# Thoracolumbar injuries: non operative treatment: indications, management

**DOI:** 10.1007/s00068-024-02619-3

**Published:** 2024-09-27

**Authors:** Christoph Nau, Hans Christoph Pape, Marko Jug, Klaus Wendt, Radko Komadina, Frank Bloemers

**Affiliations:** 1 Department of Trauma and Orthopaedic Surgery, University Hospital Frankfurt, Theodor-Stern-Kai 7, 60590 Frankfurt am Main, Germany; 2 University Hospital of Zürich, University of Zürich, Zürich, Switzerland; 3 University Medical Centre Ljubljana, University of Ljubljana, Ljubljana, Slovenia; 4 University Medical Center Groningen, University of Groningen, Groningen, The Netherlands; 5 Medical Faculty, University of Ljubljana, Ljubljana, Slovenia; 6 Amsterdam University Medical Centre, Vrije Universiteit Amsterdam, Amsterdam, The Netherlands

**Keywords:** Thoracolumbar spine injuries, Non-operative treatment, Conservative management, Fracture stability, Morphological modifiers, Functional therapy

## Abstract

**Introduction:**

Thoracolumbar spine injuries can result from various traumatic events such as falls, motor vehicle accidents, and sports injuries. While surgical intervention is often indicated for complex fractures and in case of neurological deficits, non-operative treatment remains a viable option for certain types of injuries.

**Aims:**

This manuscript aims to provide a comprehensive overview of the specific indications and treatment options of non-operative thoracolumbar spine injuries. It seeks to provide evidence-based recommendations for selecting patients suitable for conservative management based on fracture type and stability, absence of neurological deficits, spine deformity, integrity of the posterior ligament complex and patient specific factors.

## Introduction

The thoracolumbar spine is particularly susceptible to injuries caused by trauma such as falls, traffic accidents and sporting activities. The reason for this is the high leverage and impact force of the rigid thoracic spine on the flexible lumbar spine. These injuries pose a considerable medical challenge and require careful diagnosis and a differentiated therapeutic approach. While surgical interventions are often necessary for serious injuries, conservative therapy also plays an important role in clinical practice.

The decision whether a fracture of the thoracolumbar spine can be treated conservatively must be based on a variety of criteria. Biological age, bone quality, activity level, individual requirements of the patient must be considered, plus the stability of the fracture as most important criterion [1].

A fracture is defined as stable if no neurological aggravation and no change in position are to be expected in the context of functional therapy. A fracture can be described as highly unstable if mobilization threatens a neurological aggravation [2].

This article provides a comprehensive overview of the non-operative approaches employed in the management of thoracolumbar spine injuries, including patient selection, assessment, and various conservative interventions.

### Indications for non-operative therapy

Non-operative treatment should be performed if there are either general or local contraindications to surgery. These include serious internal concomitant diseases associated with a greatly increased surgical risk, as well as local reasons such as multisegmental metastases, which make sufficient stabilization impossible, or skin changes that pose a significantly higher risk of infection. In principle, as already mentioned, injuries in which there is no threat of a relevant deformity in the further course can be treated conservatively. The main indications for non-operative treatment are listed below:

1. Type of fracture and stability.

Compression fractures: These fractures usually affect the anterior column of the vertebral body and are often stable. They can usually be treated conservatively, especially if the loss of height of the spine is minimal and the posterior wall is not affected.

Stable burst fractures: Burst fractures with minimal dislocation of the bone fragments and without significant impairment of the spinal stability can be treated conservatively. Stability is often assessed using imaging techniques such as CT and/or MRI to determine the extent of vertebral and ligamentous injury [3–6].

2. Absence of neurological deficits.

Patients who do not present neurological symptoms such as sensorimotor deficits or loss of bowel or bladder control are good candidates for non-surgical treatment. The absence of neurological impairment suggests that the spinal cord and nerve roots are not significantly affected by the injury [7].

3. Deformity.

A kyphotic deformity of less than 20 degrees and a scoliotic deformity of less than 10 degrees is usually considered acceptable for conservative treatment [1].

4. Intact posterior ligament complex.

The integrity of the posterior ligamentous complex (PLC) is critical for spinal stability. Non-surgical treatment is more likely if the PLC is intact, as it helps maintain overall spinal stability [8–10].

5. Patient-specific factors.

General health and comorbidities.

Age and bone quality.

6. Patients who wish to undergo conservative treatment.

### Morphological modifiers (MM) according to the German Society of Orthopaedics and Trauma (DGOU)

Precise assessment of a fracture is crucial for optimal therapy [11]. According to the German Society of Orthopaedics and Trauma (DGOU) four morphological modifiers (MM) were introduced in addition to the AO Spine Classification [12, 13], derivable from conventional X-ray images and CT. These criteria clarify statements about the stability of the fracture and allow the possible treatment options to be derived [2, 14].

**Fig. 1:**
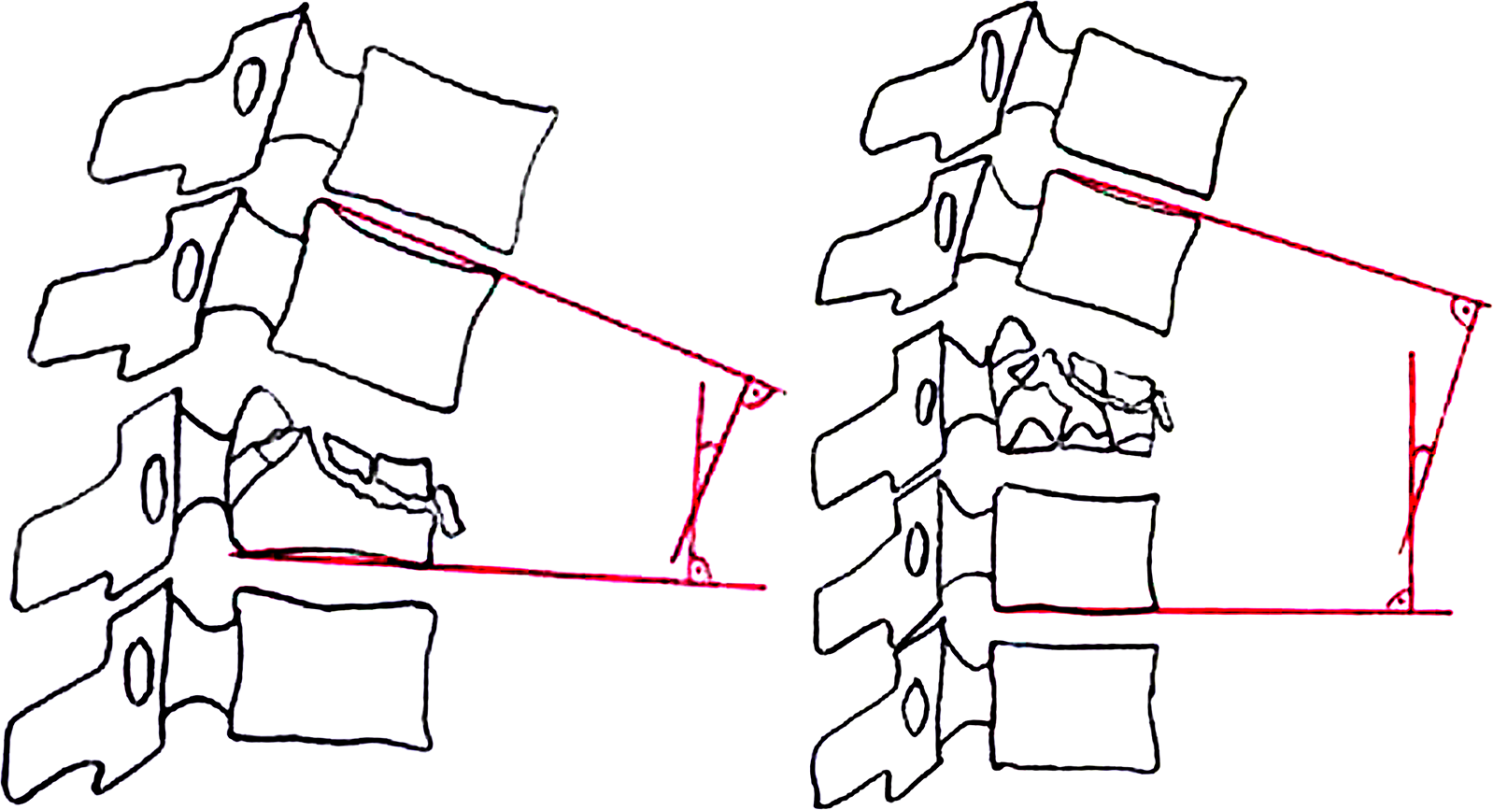
Morphological modifier 1 (MM 1): Disorder in the physiological alignment of the vertebral column: monosegmental and bisegmental endplate angle (EPA). (Spine Section DGOU [14])

**MM 1**: Deviation from the physiological profile of the spine: Fractures can affect the physiological profile of the spine both in the sagittal plane (kyphosis/lordosis) and in the frontal plane (scoliosis). To describe this deviation, monosegmental and bisegmental endplate angles (EPA) are used in the sagittal plane. The monosegmental and bisegmental scoliosis angles are used for description in the frontal plane [15, 16]. If the endplate of the injured vertebral body is involved, the bisegmental endplate angle (EPA) is used (Fig. 1).

Accordingly, the monosegmental scoliosis angle is used to describe changes in the frontal plane. This angle is formed by a straight line through the lower endplate of the injured vertebral body and through the upper endplate of the vertebral body above the injured vertebra. If the lower endplate of the injured vertebral body is involved, the bisegmental scoliosis angle should be used.

Decisive for the therapy is not only the measured angle, but the deviation from the individual sagittal profile of the spine. For this reason, the difference between the physiological angle of curvature of the spine and the measured EPA is given as δ-EPA. It should be taken into account that the EPA can differ greatly between images taken in a standing and lying position. Whenever possible, images should be taken in the standing position. If a highly unstable fracture is suspected, initial standing radiographs are not recommended. The δ-EPA at the start of therapy allows conclusions to be drawn about the stability of the fracture and the therapy options. If a δ-EPA of less than 15°- 20° is present at the start of therapy in the standing position and the posterior column is intact, no increase in the deviation from the individual sagittal profile to values requiring correction is to be expected with functional therapy. Functional therapy can therefore be a treatment option.

**Fig. 2:**
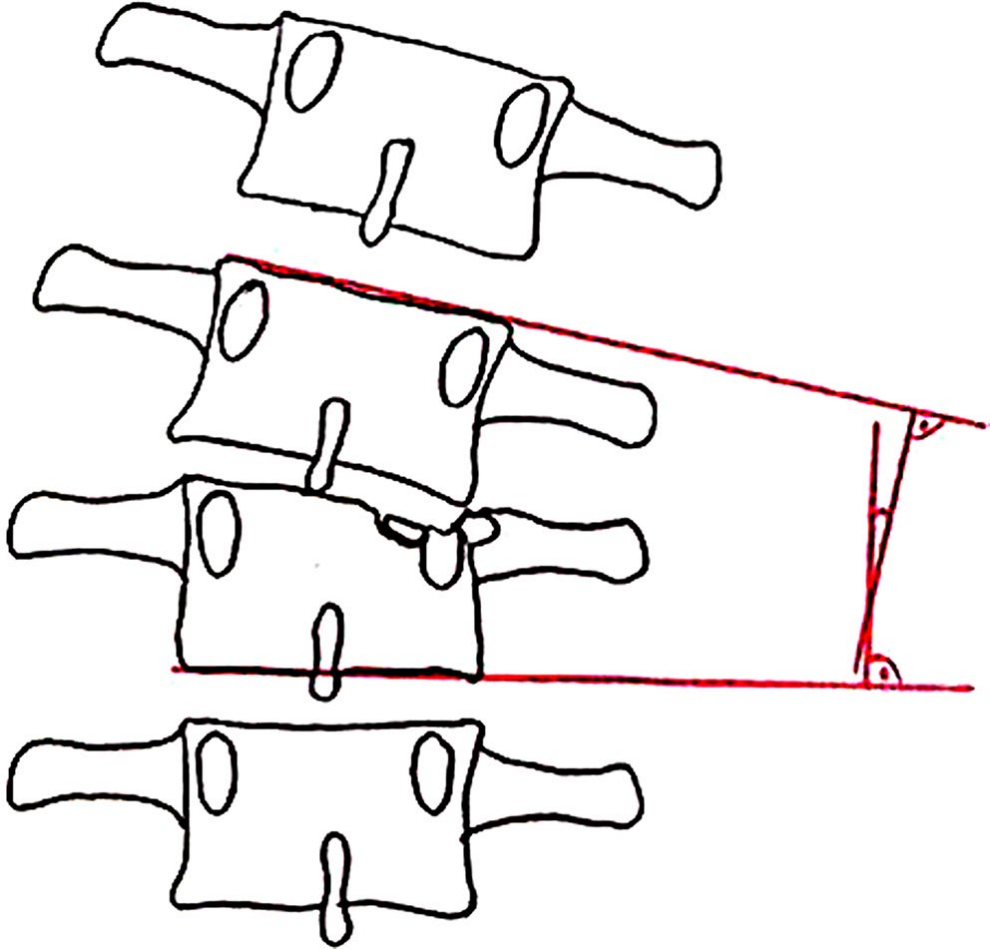
Morphological modifier 1 (MM 1): Disorder in the physiological alignment of the vertebral column: scoliosis angle. (Spine Section DGOU [14])

If there is a scoliosis angle of less than 10° at the beginning of therapy while standing, no increase in the deviation from the individual profile of the spine to values requiring correction is to be expected with functional therapy. Functional therapy can thus be a therapy option (Fig. 2).

**MM2**: Destruction of the vertebral body: The decision for surgical or conservative therapy and especially for ventral reconstruction is largely made on the basis of the destruction of the vertebral body [17–19]. The destroyed volume of the vertebral body and the fracture dislocation are significant here. To assess the destroyed volume, the vertebra is divided into three equally large, horizontal thirds. A cranial, a medium and a caudal third are distinguished. It is described to what extent the volume of the vertebral body is affected by the fracture.

**Fig. 3:**
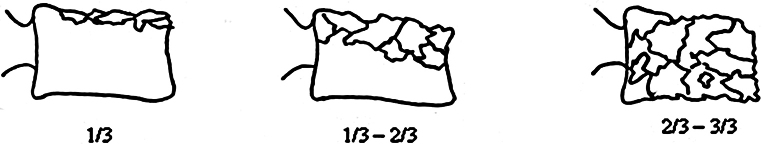
Morphological modifier II (MM II): Comminution of the vertebral body (Spine Section DGOU [14])

Another morphological criterion is the dislocation of the fragments. A distinction is made between non-dislocated fragments, dislocated less than 2 mm and fragments dislocated by more than 2 mm. It is also differentiated in which part of the vertebral body the dislocation is located. The dislocation of the fragments in the end plate area is an indication of the expected damage to the adjacent intervertebral disc (Fig. 3).

**Fig. 4:**
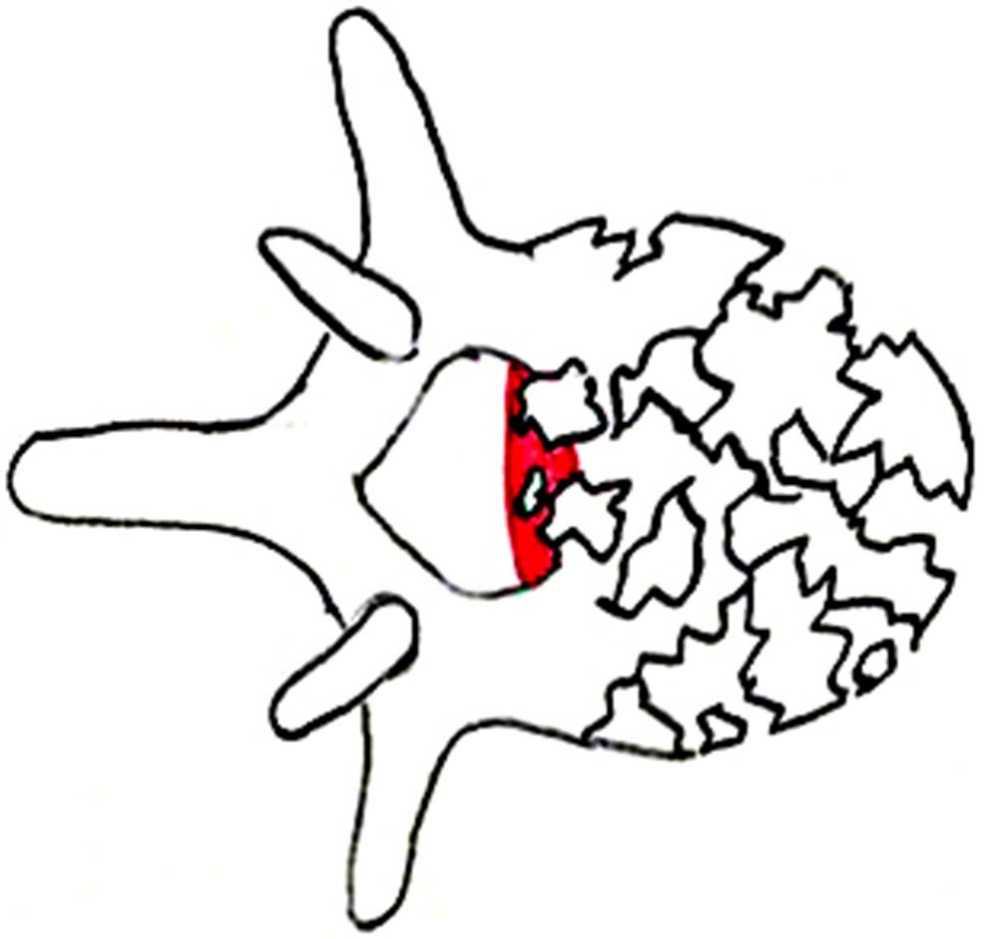
Morphological modifier III (MM III): Stenosis of the spinal canal. (Spine Section DGOU [14])

**MM 3**: Stenosis of the spinal canal: The most constricted area in the axial section of the spinal canal in the affected segment is decisive for the stenosis of the spinal canal due to bony fragments or protrusion of the posterior wall of the vertebral body. The spinal canal’s cross-sectional area is estimated in a horizontal CT section in relation to the upper and lower neighboring segments in percent (Fig. 4).

**MM 4**: Extent of disc injury: Traumatic disc injuries do not show a sufficient spontaneous healing tendency [20]. The degree of destruction of the endplate is an indication of the extent of the intervertebral disc injury. If the extent of the disc injury is unclear, an MRI should be considered.

### Based on the AO fracture classification, the following fracture types apply for non-operative treatment

#### A0 Minor, non-structural fracture

With this type of fracture, early mobilization with adequate pain therapy and physiotherapy should be take place.

#### A1 Wedge compression (MM 1)

The decisive factor here and with A3 fractures is the extent of kyphosis. With a δEPA < 15–20°, functional therapy can be initiated. In the presence of a δEPA > 15–20°, surgical therapy in the form of instrumentation is advisable to prevent an increase in the kyphosis angle.

#### A2 Split (MM 2, MM 4)

With these fracture types, early mobilization with adequate pain therapy and physiotherapy is possible. An indication for surgery may be a wide separation of the fragments and/ or lesion of the adjacent intervertebral disc [18].

#### A3 incomplete burst (MM 1, MM 2, MM3, MM 4)

With this type of fracture too, the extent of the kyphosis angle is crucial. δEPA < 15–20° and/or scoliosis < 10° can be treated functionally.

#### A4 complete burst (MM 1, MM 2, MM 3, MM 4)

The same criteria apply here as for A3 fractures.

Although A3 and A4 fractures can be treated conservatively there is a relevant risk of fragment dislocation towards the spinal canal. Therefore a lot of these fractures are treated operatively.

### Outpatient or inpatient non-surgical treatment

**Fig. 5:**
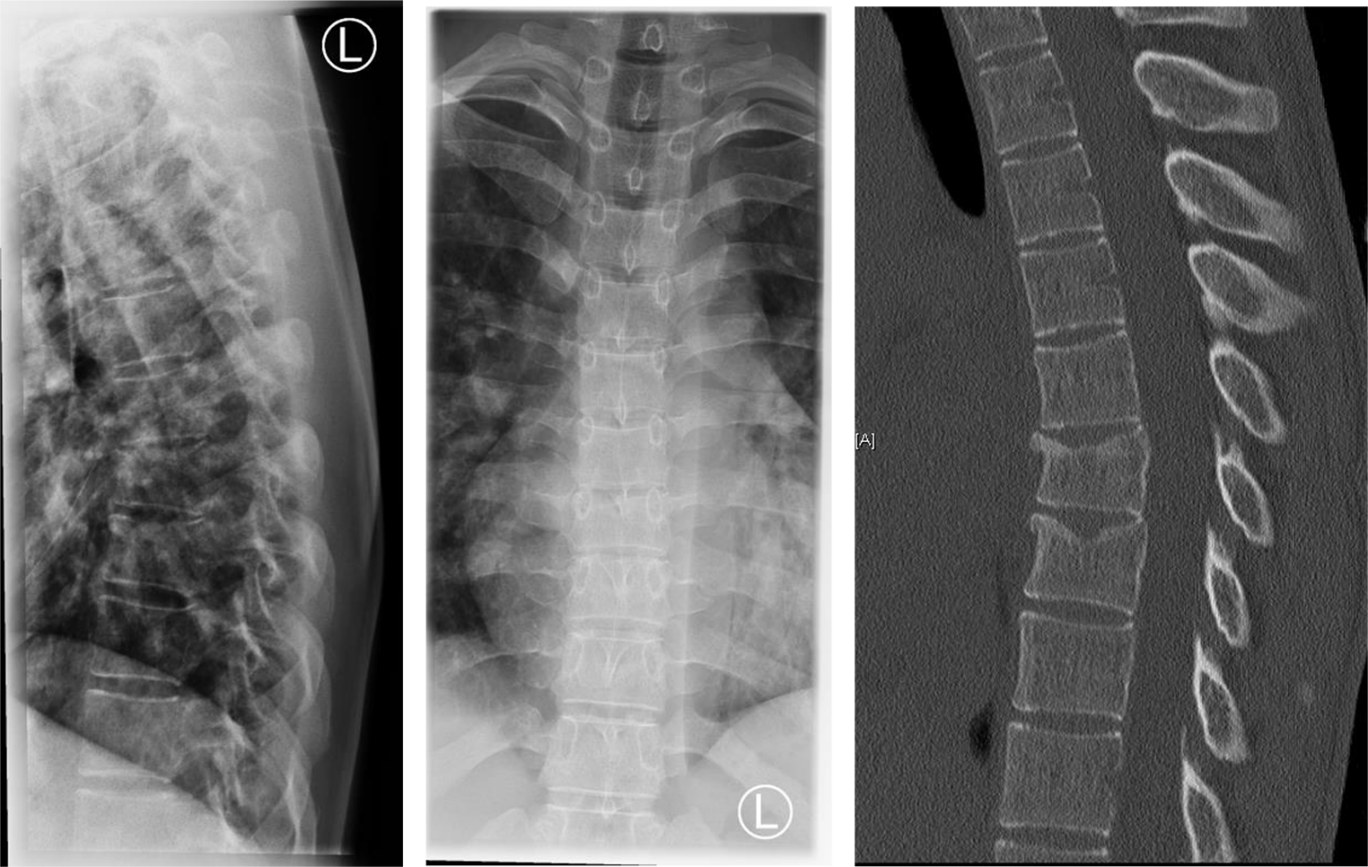
22 years, female, jump from 10 m diving board, AO type A3 fracture, Due to the age and moderate pain an MRI was not performed and conservative treatment was initiated

**Fig. 6:**
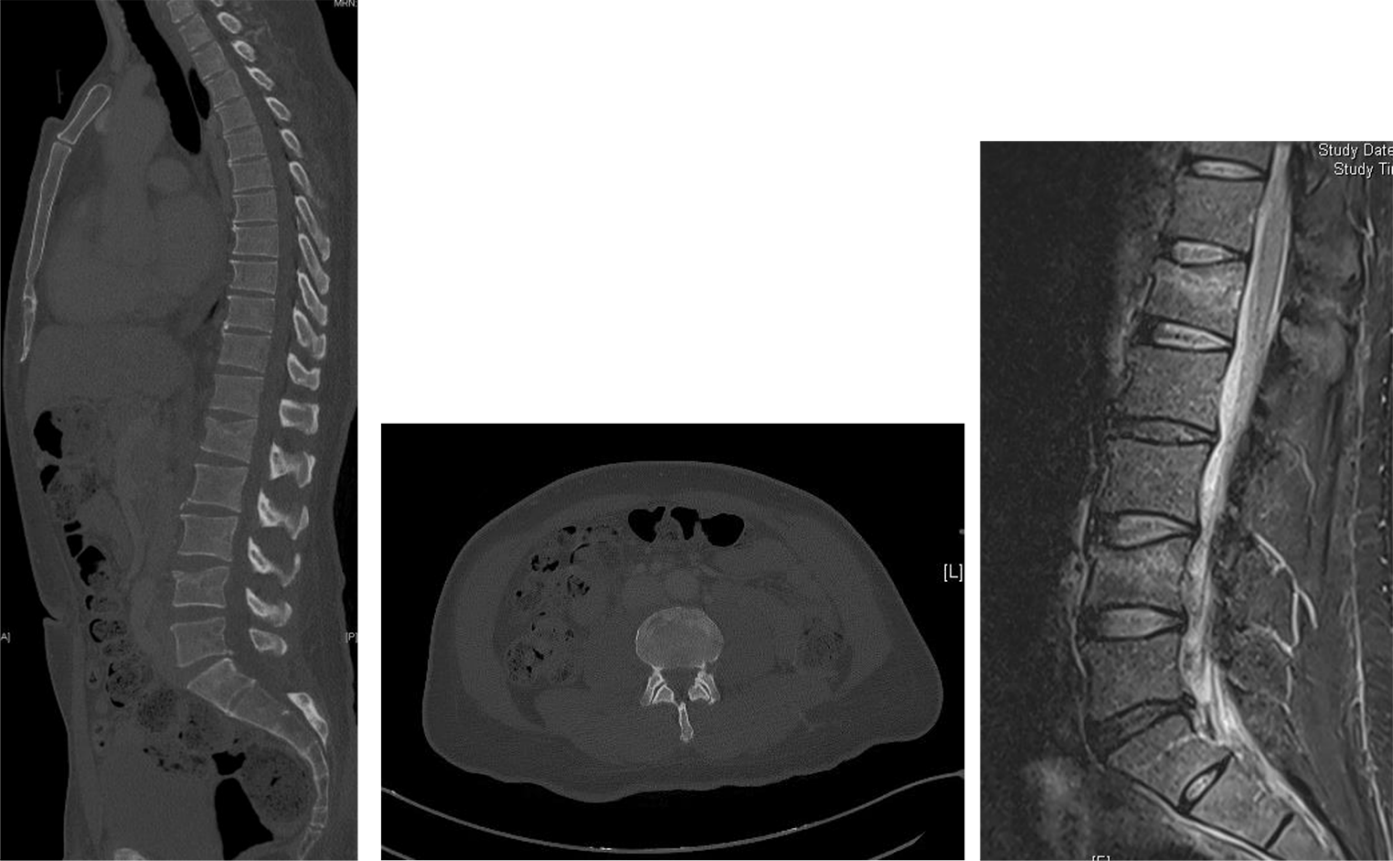
45 years, male, hit by a car as a cyclist, L1 and L4 fractures AO type A1, The adjacent discs and the posterior ligament complex are intact

**Fig. 7:**
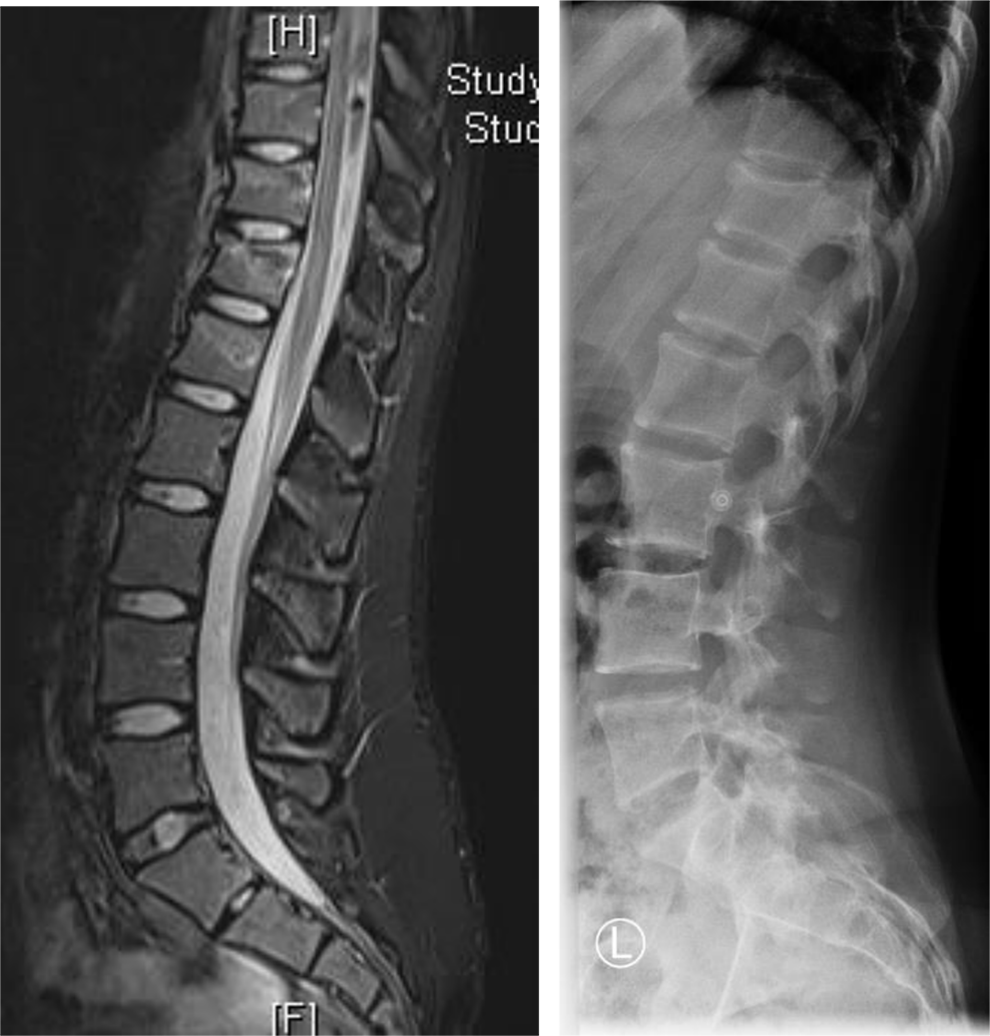
15 years, female, fall from second floor, TH 11-L1 chain injury AO type A1 L1 and A3 TH11/12, Due to her age a CT scan was not performed. Follow-up X-ray presented no further dislocation

**Fig. 8:**
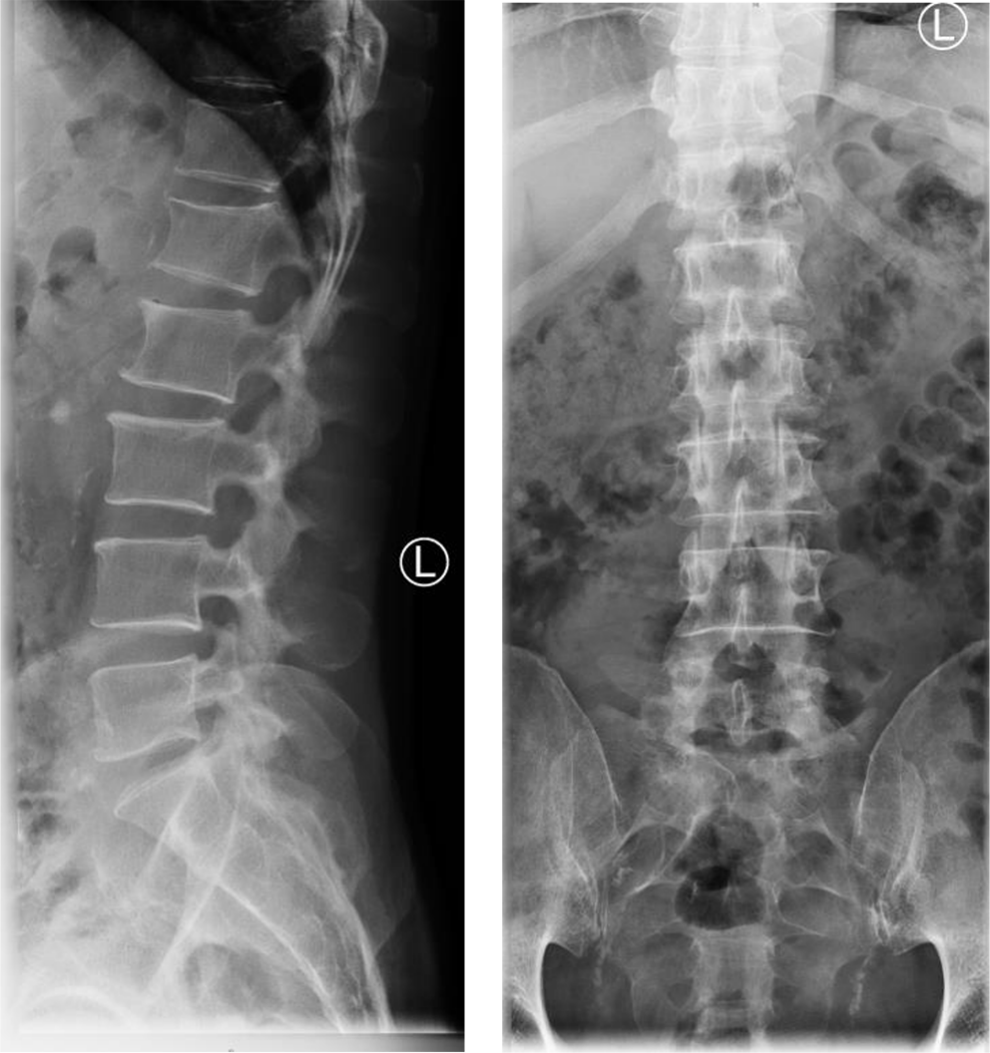
59 years, male, fall from motorcycle, TH12, L1 and L3 fractures AO type A1, clear indication for conservative treatment and moderate pain, no absolute indication for CT and MRI

Outpatient treatment of patients with conservative, thoracolumbar spinal injury is possible in cases of functional therapy, mobilized patients and properly adjusted pain therapy. Inpatient admission should occur in case of significant pain symptoms and/or insufficient mobility (Fig. 5, 6, 7 and 8).

After a short period of rest with adequate pain therapy, rapid mobilization should take place [3, 21, 22]. Clinical and radiological controls are required until fracture healing. Where possible, X-ray check- ups after 1, 3, 6 and 12 weeks should be performed while standing, as this is the only way to reliably detect malposition [23]. It may be useful to supplement these with CT or MRI. If there is an aggravation of the findings in the further course, this can lead to an indication for surgery.

Accompanying measures include sufficient thrombosis prophylaxis, physiotherapy if necessary combined with respiratory gymnastics, and decubitus prophylaxis with adequate pain therapy [24].

The question whether or not a corset should be worn has been the subject of controversial debate for many years. The results of some authors have shown no superiority of corset therapy over functional follow-up treatment without a corset in randomized, controlled studies [25]. Sometimes the use of specific orthoses can be useful [26].

The success of an non-operative therapy is based on a team approach by a close cooperation between doctor, patient, physiotherapist and nurse.

### Non-surgical treatment in children

Thoracolumbar spine injuries are relatively rare in children compared to adults and mostly affect the thoracolumbar junction. These are usually compression injuries, which can be treated conservatively in most cases. The combination of immobilization, pain management, physical therapy, and close monitoring allows many young patients to heal effectively while minimizing the risks associated with surgery [27, 28].

## Conclusion

Non-surgical treatment of thoracolumbar spinal injuries is a valid option for stable fractures without neurological deficits especially for patients with relevant pre-existing conditions. The AO spine classification in addition with the Morphological Modifiers (MM) by the German Society of Orthopaedics and Trauma (DGOU) provide an assessment of whether the fracture can be treated conservatively in a shared decision with the patient. Conservative treatment options include rapid mobilization, pain management, physiotherapy, and, in special cases the use of orthoses or braces to support the spine. However, according to the ESTES recommendations careful patient monitoring and selection with radiological controls in standing position are crucial for the success of conservative therapy (Fig. 8).

## Data Availability

No datasets were generated or analysed during the current study.
